# Nanotoxicity of ZrS_3_ Probed in a Bioluminescence Test on *E. coli* Bacteria: The Effect of Evolving H_2_S

**DOI:** 10.3390/nano10071401

**Published:** 2020-07-18

**Authors:** Olga V. Zakharova, Alexander A. Gusev, Jehad Abourahma, Nataliia S. Vorobeva, Dmitry V. Sokolov, Dmitry S. Muratov, Denis V. Kuznetsov, Alexander Sinitskii

**Affiliations:** 1 Research Institute for Environmental Science and Biotechnology, Derzhavin Tambov State University, Tambov 392000, Russia; olgazakharova1@mail.ru; 2 Department of Functional Nanosystems and High-Temperature Materials, National University of Science and Technology “MISIS”, Moscow 119991, Russia; triplplay@bk.ru (D.V.S.); muratov@misis.ru (D.S.M.); dk@misis.ru (D.V.K.); 3 Department of Chemistry, University of Nebraska—Lincoln, Lincoln, NE 68588, USA; jabourahma@huskers.unl.edu (J.A.); nataliia.vorobeva@huskers.unl.edu (N.S.V.); 4 Nebraska Center for Materials and Nanoscience, University of Nebraska—Lincoln, Lincoln, NE 68588, USA

**Keywords:** zirconium trisulfide, nanotoxicity, antibacterial properties, bioluminescence test, *Escherichia coli*

## Abstract

Materials from a large family of transition metal trichalcogenides (TMTCs) attract considerable attention because of their potential applications in electronics, optoelectronics and energy storage, but information on their toxicity is lacking. In this study, we investigated the toxicity of ZrS_3_, a prominent TMTC material, toward photoluminescent *E. coli* bacteria in a bioluminescence test. We found that freshly prepared ZrS_3_ suspensions in physiological saline solution with concentrations as high as 1 g/L did not exhibit any toxic effects on the bacteria. However, ZrS_3_ suspensions that were stored for 24 h prior to the bioluminescence tests were very toxic to the bacteria and inhibited their emission, even at concentrations down to 0.001 g/L. We explain these observations by the aqueous hydrolysis of ZrS_3_, which resulted in the formation of ZrO_x_ on the surface of ZrS_3_ particles and the release of toxic H_2_S. The formation of ZrO_x_ was confirmed by the XPS analysis, while the characteristic H_2_S smell was noticeable for the 24 h suspensions. This study demonstrates that while ZrS_3_ appears to be intrinsically nontoxic to photoluminescent *E. coli* bacteria, it may exhibit high toxicity in aqueous media. The results of this study can likely be extended to other transition metal chalcogenides, as their toxicity in aqueous solutions may also increase over time due to hydrolysis and the formation of H_2_S. The results of this study also demonstrate that since many systems involving nanomaterials are unstable and evolve over time in various ways, their toxicity may evolve as well, which should be considered for relevant toxicity tests.

## 1. Introduction

In recent years, much effort has been devoted to investigating various two-dimensional materials, such as graphene, hexagonal boron nitride, transition metal dichalcogenides, MXenes and many others [1,2,3]. Many of these materials show promise for a great variety of applications in numerous fields, including electronics, optoelectronics, composites and energy storage. With the increasing prospect of incorporation of 2D materials in very diverse consumer applications, a great deal of recent research has been focused on the assessment of their potential toxicity [4]. Understanding of the potential toxic effects associated with 2D materials is also important in the view of their widely discussed biomedical applications, which include bioimaging [5], drug delivery [6], phototherapy [7], biosensors [8] and many others.

**Figure 1:**
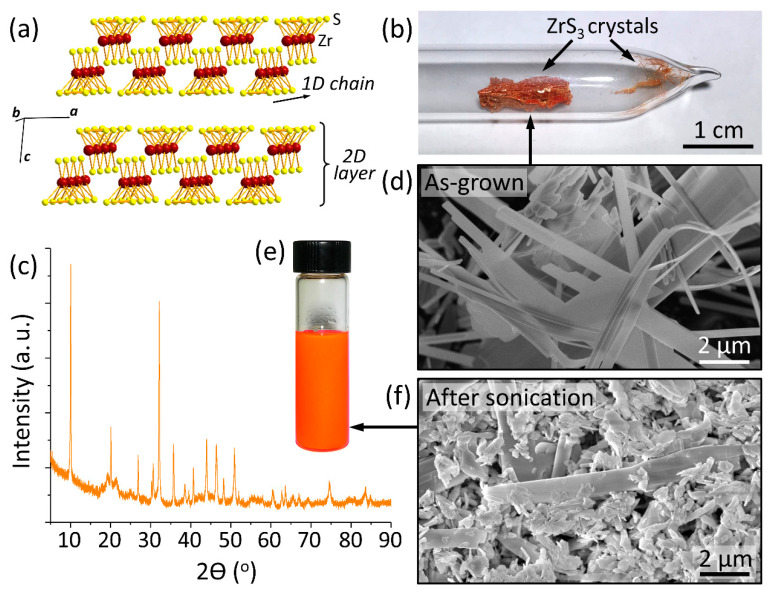
(**a**) Crystal structure of ZrS_3_ (*P* 2_1_/*m* space group); (**b**) optical photograph of an evacuated quartz ampule with orange ZrS_3_ crystals indicated by the arrows; (**c**) XRD spectrum of ZrS_3_ crystals; (**d**) SEM image of the as-grown ZrS_3_ crystals shown by the arrow in panel b; (**e**) optical photograph of a vial with 1 g/L suspension of exfoliated ZrS_3_ crystals, which were dispersed in PS solution by sonication; (**f**) SEM image of ZrS_3_ particles after sonication in PS solution.

While a considerable number of in vitro and in vivo studies have focused on the assessment of toxicity of graphene-based materials [9,10], other 2D materials have received much less attention from researchers. For example, there have been no nanotoxicity studies of materials from a large family of transition metal trichalcogenides (TMTCs) with the general formula MX_3_ (M = Ti, Zr, Hf, Nb or Ta; X = S, Se or Te) [11,12,13,14], which have recently received much attention due to their promising electronic [15,16,17,18,19,20] and optoelectronic [21,22] and thermoelectric properties [23,24,25]. The crystal structure of TMTC materials is different from other 2D materials, which is shown in Figure 1a using ZrS_3_ as an example [11,12,13,14,26]. This structure can be viewed as composed of 1D chains of ZrS_3_ trigonal prisms, in which the Zr^4+^ centers are surrounded by the sulfide (S^2−^) and disulfide (S_2_^2−^) species. The 1D chains then assemble into 2D layers through weak van der Waals-like interactions, while the layers stack into bulk crystals (Figure 1a). Because of their highly anisotropic structure, exfoliation of TMTC crystals into both 2D layers and 1D chains is possible [26].

While no TMTC has been a subject of nanotoxicity studies, this work was specifically focused on ZrS_3_, which was shown theoretically and experimentally to be a promising material for charge transport layers in perovskite light-emitting diodes [27], battery cathodes [28], nonlinear optics [29], photovoltaics and photocatalysis [30]. Furthermore, the results of a recent angle-resolved photoluminescence study suggested that because of its highly anisotropic band structure [31] and emission properties ZrS_3_ could be potentially employed in biomedical applications [32]. The breadth of possible applications of ZrS_3_, including those potentially involving living organisms, warrants nanotoxicity studies of this emerging 2D material. Here, we investigated the effect of ZrS_3_ dispersed in physiological (PS) solution on photoluminescent *Escherichia coli* (*E. coli*) bacteria using a bioluminescence test [33,34,35,36,37].

The bioluminescence test is a highly standardized method that was employed in numerous nanotoxicity studies [33,34,35,36,37,38]. It is based on monitoring the intensity of emission of photoluminescent bacteria, such as *Vibrio fischeri*, in the presence of toxic substances. The bioluminescence intensity decreases proportionally to the inhibition of bacteria that directly correlates to toxicity. For aqueous suspensions of ZrS_3_ nanoparticles we found that their toxicity to photoluminescent *E. coli* bacteria strongly depends on the time between suspension preparation and bioluminescence test. In freshly prepared suspensions the ZrS_3_ nanoparticles did not exhibit any toxicity even at concentrations up to 1 g/L. However, if suspensions were stored for 24 h prior to the tests, toxic effects were observed at concentrations down to 0.001 g/L. We explain this observation by the slow hydrolysis of ZrS_3_ in aqueous media that is accompanied by an evolution of highly toxic H_2_S.

This study demonstrates that ZrS_3_, while initially nontoxic to photoluminescent *E. coli* bacteria, starts exhibiting appreciable toxicity over time if kept in an aqueous medium. The results of this study can likely be extended to other transition metal chalcogenides, as their toxicity in aqueous solutions may also increase over time due to hydrolysis and the formation of H_2_S. The results of this study also demonstrate that since many systems involving nanomaterials are unstable and evolve over time in various ways, their toxicity may evolve as well, which should be considered for relevant toxicity tests.

## 2. Materials and Methods

All chemicals were purchased from Sigma-Aldrich (USA) unless noted otherwise. ZrS_3_ crystals were grown by the direct reaction of stoichiometric amounts of Zr metal and S vapor in an evacuated quartz ampule at 800 °C for 48 h, as described in our previous work [39]. The crystals were characterized by X-ray diffraction (XRD) using a PANalytical Empyrean diffractometer (Netherlands) with Cu Kα radiation. Scanning electron microscopy (SEM) of ZrS_3_ crystals was performed using a FEI Nova NanoSEM 450 scanning electron microscope (USA) at the accelerating voltage of 5 kV. Raman spectroscopy was performed using a Thermo Scientific DXR Raman microscope (USA) with a 532 nm excitation laser and a 100× objective. XPS analysis was performed at room temperature using a Thermo Scientific K-Alpha X-ray photoelectron spectrometer (USA) with a monochromatic Al Kα (1486.6 eV) X-ray source and a low energy electron flood gun for charge compensation. High-resolution XPS spectra of Zr*3d* and S*2p* were collected using a pass energy of 20 eV and a 0.1 eV step. To collect XPS spectra of pristine ZrS_3_, as-grown crystals were cleaved in air directly before introduction into the loading vacuum chamber to minimize the contribution of surface contamination. XPS spectra of solution-exfoliated ZrS_3_ crystals were recorded on samples produced by drying droplets of the corresponding suspensions in air.

To prepare ZrS_3_ suspensions for nanotoxicity experiments, ZrS_3_ crystals were sonicated in physiological saline solution (PS; 9 g/L NaCl aqueous solution) for 20 min to produce a homogeneous orange suspension with a concentration of 1 g/L. Other suspensions were prepared by diluting this ZrS_3_ stock suspension to concentrations of 0.0001, 0.001, 0.01 and 0.1 g/L. The nanotoxicity experiments were performed on freshly prepared ZrS_3_ suspensions, which were analyzed less than 0.5 h after preparation and the suspensions that were stored for 24 h prior to the measurements.

The laboratory glassware for sample storage and biotesting was soaked in a mixture of potassium bichromate and sulfuric acid for about 3 h, then washed with deionized (DI) water, deacidified with a sodium bicarbonate solution and finally washed four times with DI water and dried in an oven.

The toxicity of ZrS_3_ suspensions was measured by a bioluminescence technique that is generally used for the assessment of toxic effects of nanomaterials. The procedure was similar to the widely used bioluminescence inhibition test with *Vibrio fischeri* [33,34,35,36,37]. We monitored changes in intensity of bioluminescence of genetically modified photoluminescent *E. coli* bacteria in the presence of ZrS_3_ nanoparticles or other tested chemicals compared to the emission of a control sample (same bacteria in a pure PS solution).

We used commercial recombinant *E. coli* K12 TG1 bacteria modified with *luxCDABE* genes of photoluminescent *Photobacterium leiognathi* 54D10 marine bacteria. Lyophilized *E. coli* K12 TG1 bacteria under the brand name Ecolum were purchased from NVO ImmunoTek (Russian Federation). Before the nanotoxicity experiments, the lyophilized bacteria were rehydrated in a PS solution at 4 °C for 30 min and then at room temperature for 1 h. The solutions used in this study had an *E. coli* concentration of about 2.5 × 10^7^ cells/mL. In a typical bioluminescence experiment, 0.1 mL of this *E. coli* solution was mixed with 0.9 mL of a ZrS_3_ suspension in PS. The resulting mixture was stored for 30 min prior to the measurements, which were performed using a NIKI MLT Biotox-10 luminometer (Russian Federation). The instrument detected bioluminescence of bacteria in the 300–600 nm spectral range with the maximum detector sensitivity between 380 and 490 nm. Parallel measurements of the studied and control samples were carried out. The control samples were prepared by diluting 0.1 mL of the *E. coli* stock solution with 0.9 mL of PS.

We compared the toxicity of ZrS_3_ with that of zirconyl chloride (ZrOCl_2_·8H_2_O), a common zirconium compound. Both compounds contain Zr^4+^, but while ZrS_3_ is practically insoluble in water and was present in the PS suspensions as partially exfoliated particles, zirconyl chloride is water soluble. We prepared 0.000172, 0.00172, 0.0172, 0.172 and 1.72 g/L solutions of ZrOCl_2_·8H_2_O in PS, which matched the 0.0001, 0.001, 0.01, 0.1 and 1 g/L ZrS_3_ suspensions, respectively, in zirconium concentrations. Then, 0.9 mL aliquots of zirconyl chloride solutions were mixed with 0.1 mL aliquots of the *E. coli* stock solution, and the resulting mixtures were stored for 30 min and used in bioluminescence experiments.

For the positive control we used sodium dichloroisocyanurate (C_3_Cl_2_N_3_NaO_3_, SDC), a compound with well-known toxicity that is widely used as an efficient disinfectant against Gram-positive and Gram-negative bacteria, viruses and fungi [40]. We prepared 0.0001, 0.001, 0.01, 0.1 and 1 g/L solutions of SDC in PS, and then, 0.9 mL aliquots of these solutions were mixed with 0.1 mL aliquots of the *E. coli* stock solution. The resulting mixtures were stored for 30 min and used in bioluminescence experiments. As in the experiments with ZrS_3_, the bioluminescence tests with ZrOCl_2_ and SDC involved control samples prepared by diluting 0.1 mL aliquots of the *E. coli* stock solution with 0.9 mL aliquots of PS.

Each bioluminescence experiment was performed at least 5 times, and the averaged results are presented.

## 3. Results and Discussion

Figure 1b shows an optical photograph of a reaction ampule after the ZrS_3_ growth, which was performed through a direct reaction between a Zr foil and sulfur vapor at 800 °C. While some crystals form on the quartz surface (Figure 1b), most of the ZrS_3_ grows directly on a foil forming a spongy material comprising disordered ZrS_3_ nanobelt crystals. This material was highly crystalline, as illustrated by the XRD spectrum in Figure 1c. The peaks in the XRD spectrum are narrow and sharp, and their positions agree well with prior literature reports for ZrS_3_ crystals [27,31,39,41]. A representative SEM image of the ZrS_3_ crystals is shown in Figure 1d. The crystals had a nanobelt shape, which reflects their quasi-1D crystal structure (Figure 1a) with the *b* crystallographic direction of 1D chains corresponding to the long axes of the crystals. Most of the as-grown ZrS_3_ crystals were over 10 μm long. Figure 1e shows an optical photograph of a uniform orange suspension of exfoliated ZrS_3_ crystals in PS (1 g/L) that was used for the toxicity experiments. After the sonication, the average crystal size decreased, which is illustrated by the SEM image in Figure 1f (note that the SEM images of ZrS_3_ crystals before and after sonication are shown in panels d and f, respectively, at the same magnification). While some ZrS_3_ crystals retained their nanobelt shape, others were split by sonication into randomly shaped particles with sizes of about 1 μm.

**Figure 2:**
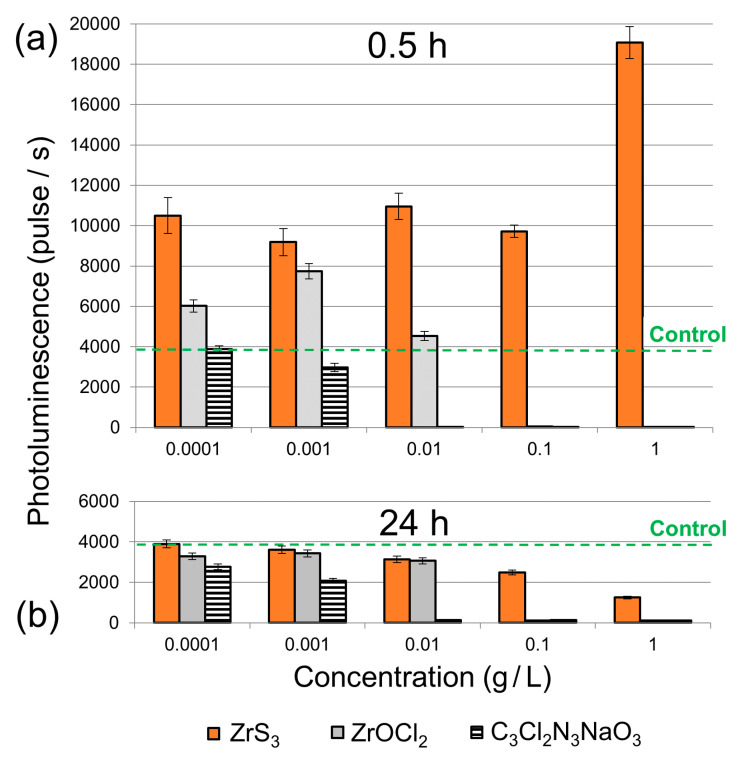
*E. coli* luminescence values in (**a**) as-prepared (0.5 h) and (**b**) 24 h solutions of ZrS_3_, ZrOCl_2_ and C_3_Cl_2_N_3_NaO_3_. For ZrOCl_2_ solutions, the concentrations were 0.000172, 0.00172, 0.0172, 0.172 and 1.72 g/L, so that they matched the 0.0001, 0.001, 0.01, 0.1 and 1 g/L ZrS_3_ suspensions in the zirconium concentrations, respectively.

The results of bioluminescence experiments are summarized in Figure 2. In both panels the emission of the control sample (*E. coli* in a pure PS solution) is indicated by the green dashed lines at 3754 pulses/s. As expected, SDC exhibited a strong toxic effect on *E. coli* bacteria, which showed decreased photoluminescence compared to the control sample for all SDC concentrations (Figure 2). The decrease in the bacterial activity was proportional to the SDC concentration.

For the zirconium compounds compared in this study, ZrS_3_ was generally less toxic than ZrOCl_2_ at all concentrations, regardless of whether the solutions were stored for 30 min or 24 h prior to their mixing with *E. coli* bacteria. In both cases, the ZrOCl_2_ solutions strongly suppressed the bacterial activities at concentrations above 0.01 g/L (Figure 2). The higher toxicity of ZrOCl_2_ compared to ZrS_3_, especially for the freshly prepared solutions (Figure 2a), can be explained by its solubility in aqueous media. Even though all these samples contained same amounts of zirconium, in the solutions of water-soluble ZrOCl_2_ all Zr(IV) was available for interaction with the bacteria in a form of aqueous complexes. On the contrary, since ZrS_3_ is insoluble in water most Zr(IV) in the suspensions was in the bulk of ZrS_3_ particles and thus did not directly interact with the bacteria.

The toxic effects were very different for the ZrS_3_ suspensions that were stored for 30 min or 24 h prior to their mixing with the bacteria. Freshly prepared ZrS_3_ suspensions did not exhibit any toxicity to the photoluminescent *E. coli* bacteria across the entire tested concentration range (Figure 2a). In fact, for all studied concentrations we observed a considerable stimulating activity of ZrS_3_ nanoparticles on the bioluminescence of *E. coli*, as all emission values were higher than for the control sample at 3754 pulses/s. However, the ZrS_3_ suspensions that were stored for 24 h prior to the experiment suppressed the bioluminescence of *E. coli* even at a concentration as low as 0.001 g/L (Figure 2b), indicating their high toxicity.

**Figure 3:**
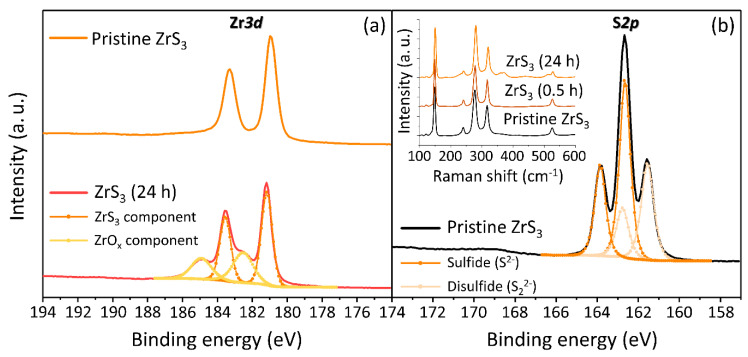
Spectroscopic characterization of ZrS_3_ crystals before and after sonication. (**a**) Comparison of XPS Zr*3d* spectra of pristine ZrS_3_ crystals (top) and ZrS_3_ nanoparticles stored in water for 24 h (bottom); (**b**) XPS S*2p* spectrum of pristine ZrS_3_. Inset shows Raman spectra of pristine ZrS_3_ crystals, ZrS_3_ nanoparticles from a freshly prepared suspension (0.5 h), and ZrS_3_ nanoparticles stored in an aqueous medium for 24 h.

It should be noted that the ZrS_3_ suspensions that were stored for 24 h produced a mild but noticeable smell of H_2_S, which can form as a result of aqueous hydrolysis of ZrS_3_. Because of its well-known toxic effects, hydrogen disulfide forming in aqueous ZrS_3_ suspensions over time could be responsible for their increased toxicity. Since the hydrolysis of ZrS_3_ is accompanied by the formation of zirconium oxide (ZrO_x_) on the surface of nanoparticles, we investigated this process using XPS (Figure 3).

Figure 3a shows XPS Zr*3d* spectra of pristine ZrS_3_ crystals and the ZrS_3_ nanoparticles stored in an aqueous medium for 24 h. The top spectrum shows two peaks located at 181.16 eV and 183.55 eV, both of which can be assigned to ZrS_3_ [42]. The XPS Zr*3d* spectrum of the ZrS_3_ nanoparticles stored in an aqueous medium can be deconvoluted into two doublet peaks. In addition to the same doublet found in pristine ZrS_3_, the bottom spectrum in Figure 3a also shows the Zr*3d*_3/2_ and Zr*3d*_5/2_ components of ZrO_x_ at 182.52 eV and 184.95 eV, respectively. The spin-orbit doublet splittings for ZrS_3_ and ZrO_x_ are 2.39 eV and 2.43 eV, respectively [43]. Thus, these spectra confirm the formation of ZrO_x_ on the surface of ZrS_3_ nanoparticles. The XPS S*2p* spectrum contains signals from sulfur located in the sulfide (S^2−^) and disulfide (S_2_^2−^) species. The spectrum shown in Figure 3b was fitted using two doublet peaks. The binding energies of S*2p*_1/2_ and S*2p*_3/2_ for the sulfide are 161.55 eV and 162.65 eV, respectively. The disulfide group peaks are located at 162.76 eV and 163.85 eV. The intensity ratio of two sulfur components is a characteristic value for ZrS_3_ and should be equal to 2. The calculated ratio of the fitted S_2_^2−^/S^2−^ peaks is 1.85, which is in good agreement with literature data [42].

We also compared the as-grown ZrS_3_ crystals with the solution-exfoliated ZrS_3_ nanoparticles by Raman spectroscopy, see the inset in Figure 3b. The Raman spectrum of pristine ZrS_3_ crystals confirms their high quality and crystallinity, showing very sharp and well-resolved peaks at about 148, 277, 317 and 526 cm^−1^ in accordance with previous studies [32,44]. The spectrum of ZrS_3_ nanoparticles from a freshly prepared suspension is nearly identical to that of the as-grown crystals, demonstrating that while the sonication decreased the average size of particles (see Figure 1d,f), their structure and crystallinity were not much affected. The Raman spectrum of ZrS_3_ nanoparticles from a suspension that was stored for 24 h prior to the measurements also looks very similar to the other two spectra (see the inset in Figure 3b). While XPS, a surface-sensitive method, demonstrated the formation of ZrO_x_ on the surface of ZrS_3_ nanoparticles, Raman spectroscopy shows that in bulk they retained their properties.

The XPS results confirm the formation of ZrO_x_ due to the aqueous hydrolysis of ZrS_3_, which is accompanied by the evolution of H_2_S. It is plausible that toxic H_2_S is responsible for the increased toxicity of ZrS_3_ suspensions stored for 24 h (Figure 2b). The increased bioluminescence observed for the freshly prepared ZrS_3_ suspensions could also be related to the formation H_2_S that is initially formed in very small quantities. Other studies reported that low concentrations of hydrogen disulfide may exhibit stimulation of bacteria and protect them against oxidative stress [45,46], which is a common toxicity mechanism for nanoparticles. As the H_2_S concentration increases over the 24 h storage period, the ZrS_3_ suspensions become highly toxic to bacteria, which we observed experimentally.

## 4. Summary

This work reports the first nanotoxicity study of materials from a large TMTC family. More specifically, we investigated the toxicity of ZrS_3_, a prominent TMTC material, toward photoluminescent *E. coli* bacteria in a bioluminescence test. We investigated the solution exfoliation of ZrS_3_ and prepared aqueous suspensions of ZrS_3_ nanoparticles with concentrations ranging from 0.0001 to 1 g/L. We found that freshly prepared ZrS_3_ suspensions with concentrations as high as 1 g/L did not exhibit any toxic effects on the bacteria and, on the contrary, stimulated their activity. However, ZrS_3_ suspensions that were stored for 24 h prior to the bioluminescence tests were very toxic to the bacteria and inhibited their emission even at concentrations down to 0.001 g/L. We explain these observations by the aqueous hydrolysis of ZrS_3_, which resulted in the formation of ZrO_x_ on the surface of nanoparticles and the release of toxic H_2_S. The formation of ZrO_x_ was confirmed by the XPS analysis, while the characteristic H_2_S smell was noticeable for the 24 h suspensions. Hydrogen disulfide could be responsible for the bacterial stimulation in the freshly prepared ZrS_3_ suspensions, because at small concentrations H_2_S was shown to protect bacteria against oxidative stress [45,46], which is widely regarded as a common nanoparticle-induced toxicity mechanism. However, as more H_2_S was released during the hydrolysis of ZrS_3_ over a 24 h period, the suspensions became toxic to the bacteria.

This study demonstrates that ZrS_3_ suspensions, while initially nontoxic to photoluminescent *E. coli* bacteria, start exhibiting appreciable toxicity over time. The results of this study can likely be extended to other transition metal chalcogenides, as their toxicity in aqueous solutions may also increase over time due to hydrolysis and the formation of H_2_S. The results of this study also demonstrate that since many systems involving nanomaterials are unstable and evolve over time in various ways, their toxicity may evolve as well, which should be considered for relevant toxicity tests.
